# Organizing principles of whole-brain functional connectivity in zebrafish larvae

**DOI:** 10.1162/netn_a_00121

**Published:** 2020-03-01

**Authors:** Richard F. Betzel

**Affiliations:** Department of Psychological and Brain Sciences, Indiana University, Bloomington, IN, USA; Cognitive Science Program, Indiana University, Bloomington, IN, USA; Program in Neuroscience, Indiana University, Bloomington, IN, USA; IU Network Science Institute, Indiana University, Bloomington, IN, USA

**Keywords:** Mesoscale connectomics, Functional connectivity, Modularity, Wiring cost, Flexibility

## Abstract

Network science has begun to reveal the fundamental principles by which large-scale brain networks are organized, including geometric constraints, a balance between segregative and integrative features, and functionally flexible brain areas. However, it remains unknown whether whole-brain networks imaged at the cellular level are organized according to similar principles. Here, we analyze whole-brain functional networks reconstructed from calcium imaging data recorded in larval zebrafish. Our analyses reveal that functional connections are distance-dependent and that networks exhibit hierarchical modular structure and hubs that span module boundaries. We go on to show that spontaneous network structure places constraints on stimulus-evoked reconfigurations of connections and that networks are highly consistent across individuals. Our analyses reveal basic organizing principles of whole-brain functional brain networks at the mesoscale. Our overarching methodological framework provides a blueprint for studying correlated activity at the cellular level using a low-dimensional network representation. Our work forms a conceptual bridge between macro- and mesoscale network neuroscience and opens myriad paths for future studies to investigate network structure of nervous systems at the cellular level.

## Author Summary

Little is known about the principles by which mesoscale functional networks are organized and whether they parallel the features of macroscale networks. Here, we used network science methods to investigate the architecture of functional connectivity in zebrafish larvae. We find that its architectural features are remarkably similar to that of macroscale functional brain networks, with connection weights exhibiting clear distance-dependence, evidence of multiscale and hierarchical community structure, high participation hub regions, and flexible reconfiguration across a range of tasks.

## INTRODUCTION

Nervous systems are collections of functionally and structurally connected neurons, neuronal populations, and brain areas (Sporns, Tononi, & Kötter, 2005). Coordination of and within these networks underpins an organism’s ability to process sensory stimuli (Downar, Crawley, Mikulis, & Davis, 2000; Ko et al., 2011), to successfully navigate its environment (Hartley, Lever, Burgess, & O’Keefe, 2014; Jacobs et al., 2013), and to perform goal-directed action (Spreng, Stevens, Chamberlain, Gilmore, & Schacter, 2010). Network science provides a quantitative framework for representing and analyzing the organization of biological neural networks (Bassett & Sporns, 2017). Within this framework, neural elements and their pairwise interactions are modeled as the nodes and edges of a complex network (Bullmore & Sporns, 2009), to which one can apply a growing suite of powerful graph-theoretic tools to assay the network’s structural (Rubinov & Sporns, 2010), functional (Park & Friston, 2013), and dynamical (Deco, Jirsa, Robinson, Breakspear, & Friston, 2008) properties.

Though the network model can be applied to nervous systems imaged at virtually any spatial scale (Betzel & Bassett, 2017b; Craddock et al., 2013; Schröter, Paulsen, & Bullmore, 2017), the majority of applications thus far have focused on the macroscale, where nodes represent brain regions and connections represent pairwise statistical associations of recorded activity (functional connectivity; FC). Though macroscale network analyses have been most successful in linking variation of network features to cognition (Park & Friston, 2013), disease (Fornito, Zalesky, & Breakspear, 2015), and development (Di Martino et al., 2014), they have also begun to elucidate the general principles by which biological neural networks are organized (Betzel et al., 2016; Rubinov, 2016).

These principles include a balance between network structures that support segregative (local and specialized) and integrative (global and generalized) information processing (Cohen & D’Esposito, 2016; Sporns, Tononi, & Edelman, 2002), such as modules versus hubs (Meunier, Lambiotte, Fornito, Ersche, & Bullmore, 2009; Power, Schlaggar, Lessov-Schlaggar, & Petersen, 2013; Sporns & Betzel, 2016; van den Heuvel & Sporns, 2013), a strong brain-wide drive to reduce the material and metabolic cost of wiring (Betzel & Bassett, 2018; Bullmore & Sporns, 2012; B. L. Chen, Hall, & Chklovskii, 2006), and an intrinsic functional architecture that reconfigures subtly and efficiently in response to tasks or stimulation (Cole, Bassett, Power, Braver, & Petersen, 2014; Schultz & Cole, 2016).

While these organizing principles have been observed at the macroscale across individuals and phylogeny (Rubinov, Ypma, Watson, & Bullmore, 2015; van den Heuvel, Bullmore, & Sporns, 2016), it remains unclear whether analogous principles shape the architecture of nervous systems at the mesoscale, where networks represent interactions among collections of cells and molecules (Humphries, 2017; Schröter et al., 2017). Though the number of studies investigating mesoscale network structure continues to grow (Betzel, Wood, Angeloni, Geffen, & Bassett, 2018; Briggman & Kristan, 2006; Bruno, Frost, & Humphries, 2015; Dann, Michaels, Schaffelhofer, & Scherberger, 2016; Faber, Timme, Beggs, & Newman, 2018; Lee et al., 2016; Mann, Gallen, & Clandinin, 2017; Orlandi, Soriano, Alvarez-Lacalle, Teller, & Casademunt, 2013; Romano et al., 2017; Rosch, Hunter, Baldeweg, Friston, & Meyer, 2017; Yamamoto et al., 2018), technological limitations restricting the field of view and an emphasis on neuronal populations as the unit of interest (rather than the brain as a whole) have made it difficult to uncover the general principles by which mesoscale functional networks are organized.

Currently, the organizing principles underpinning whole-brain mesoscale networks remain largely unexplored. Here, we take advantage of recent technological advances (Ahrens et al., 2012; Ahrens, Orger, Robson, Li, & Keller, 2013; Keller & Ahrens, 2015; Panier et al., 2013; Vladimirov et al., 2014) and a publicly available dataset (X. Chen et al., 2018) to investigate spontaneous and stimulus-evoked FC in zebrafish larvae. Our analyses reveal several putative organizing principles. These include strong geometric constraints on the magnitude and valence of connection weights, and evidence of hierarchical and multiscale modular structure balanced by the presence of polyfunctional hubs. We show that spontaneous and stimulus-evoked networks are highly similar. Nonetheless, we also find evidence of stimulus-driven module reconfiguration. Interestingly, the nodes with the greatest propensity for reconfiguration overlap with the same polyfunctional hubs uncovered under spontaneous conditions, linking intrinsic network architecture to behavior. In summary, our findings link whole-brain macro- and microscale analyses and highlight network science as a framework for bridging neuroscientific inquiry across spatial scales and domains.

## RESULTS

Here, we aimed to uncover organizing principles of spontaneous and stimulus-evoked FC in zebrafish larvae. All details concerning data acquisition and network construction are included in the **Materials and Methods** section. The following subsections are organized as follows. First, we analyze group-representative spontaneous FC to identify signatures of geometric constraints, hierarchical modular structure, and polyfunctional hubs. Next, we extend these analyses to stimulus-evoked FC and show that it is shaped by the brain’s intrinsic (spontaneous) functional organization. Finally, we present results of single-subject analyses and show a high level of intersubject consistency.

### Geometric Organization of Spontaneous FC

**Figure 1.**
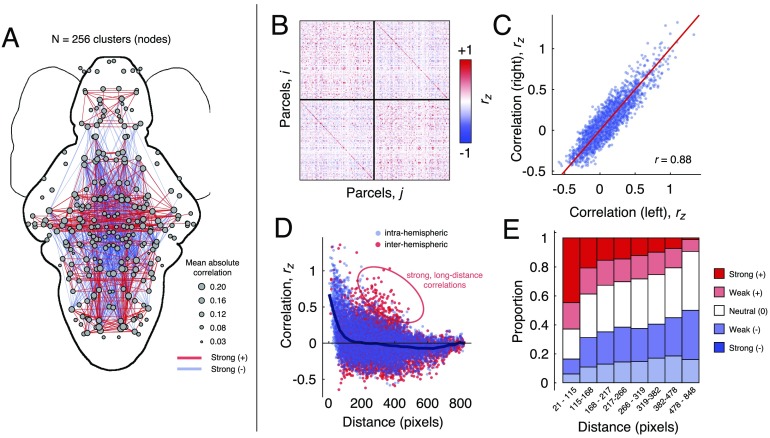
Spatial features of zebrafish whole-brain functional connectivity. (A) Thresholded network in anatomical space. Nodes represent parcels with size proportional to average absolute correlation. Red and blue lines represent top 400 positive and negative correlations/anticorrelations according to magnitude. (B) Correlation matrix, *A*, ordered by hemisphere. (C) Scatterplot of left *versus* right within-hemisphere connectivity (D) Scatterplot of straight-line distance between parcel centroids and their correlation magnitude. Within- and between-hemisphere connections are plotted separately. Note that in both cases, connection weight decays as a function of distance. However, there nonetheless exists a small fraction of long-distance interhemispheric connections. (E) Breakdown of connection types by distance bins. Note that in general, the proportion of positive (strong or weak) correlations decreases with distance, while the prevalence of anticorrelations increases with over longer distances. In this panel, the term “neutral” is used to refer to correlations whose magnitude was close to 0.

Previous analyses of whole-brain FC have revealed that connection weights and other network features are shaped by underlying geometric relationships, such as nodes’ locations in space (Bellec et al., 2006; Stiso & Bassett, 2018; Vértes et al., 2012). These constraints result in networks that favor strong, short-range connections and are believed to reflect brain-wide drives to reduce the metabolic cost of coordinating activity between brain areas over long distances (Laughlin & Sejnowski, 2003). However, it remains largely unknown whether whole-brain mesoscale functional networks are subject to similar constraints. To address this question, we analyzed single-cell calcium fluorescence traces from *N*_*sub*_ = 11 zebrafish larvae recorded in stimulus-free (i.e., spontaneous) conditions. We aggregated cells into *N* = 256 hemispherically symmetric, functionally homogeneous, and spatially localized parcels (Figure 1A; see **Materials and Methods** for details; see Supporting Information Figure S4a for a side projection of same figure). The number of cells per parcel followed a heavy-tailed distribution, with many parcels containing a small number of cells and a few parcels containing disproportionately many (See Supporting Information Figure S1). We then calculated the average fluorescence trace for each parcel (node), and computed *A* = {*A*_*ij*_}, the full matrix of Fisher-transformed Pearson correlations (Figure 1B; see **Materials and Methods** for preprocessing details). Unless otherwise noted, all subsequent analyses were carried out on this group-averaged matrix *A*, which we regarded as a fully weighted and signed connectivity matrix.

First, to assess whether FC exhibited hemispheric symmetries, we calculated the similarity of all left and right within-hemisphere connections (Figure 1C). We observed that within-hemisphere connectivity patterns were highly correlated (*r* = 0.88; *p* < 0.05). To further assess the relationship of network architecture with geometry, we then plotted connection weight as a function of the Euclidean distance between parcel centroids. We found that, on average, connection weight decayed monotonically as a function of distance. However, we also observed a small subset of interhemispheric connections that were unexpectedly strong given their length (Figure 1D). We provide an additional analysis of these stronger-than-expected connections as they relate to homotopy in Supporting Information Figure S2. A similar pattern was observed when we classified connections according to their valence and magnitude, and examined the proportion of each class within a fixed set of distance bins (Figure 1E). In general, nodes separated by short distances tended to be linked by strong, positive correlations. At longer distances, however, the proportion of positive correlations decreased and was overtaken by an increase in neutral (i.e., weak correlations whose magnitude was near zero) to strong anticorrelations.

These observations confirm that geometric relationships serve as powerful determinants of connections’ strengths and valences. Despite the fact that connection weight (on average) decreases monotonically with distance, the presence of strong, long-distance correlations suggests that geometry insufficiently explains brain-wide patterns of FC, and that coordination of activity over long distances may act as an additional functional constraint on network architecture. Collectively, these findings are analogous to those observed at the macroscale (Bellec et al., 2006; Stiso & Bassett, 2018) and draw a clear conceptual link between the organization of biological neural networks at the macro- and mesoscales.

### Modular Organization of Spontaneous FC

In the previous section, and in agreement with observations made at the macroscale, we suggested that a monotonically decaying relationship of connection weight with distance may serve as a key organizing principle responsible for shaping the architecture of biological neural networks. Here and in the next section, we explore another putative organizing principle. Namely, the requirement that biological neural networks balance features that support both segregated (localized) and integrated (global) brain function, that is, network modules and hubs, respectively (Cohen & D’Esposito, 2016; Sporns et al., 2002).

Modules are groups of nodes that are densely connected to one another, but weakly connected between groups (Meunier et al., 2009; Sporns & Betzel, 2016). Because of modules’ near-autonomy from one another, they are thought to represent groups of nodes that perform the same or similar brain function and are believed to engender specialized information processing. In general, modules are not restricted to a single topological scale and can be arranged hierarchically, with deeper levels of the hierarchy reflecting increasing functional specialization (Betzel & Bassett, 2017b; Betzel, Bertolero, Gordon, et al., 2018; Betzel et al., 2013).

While modular organization has been frequently observed in whole-brain macroscale networks (Bertolero, Yeo, & D’Esposito, 2015; Power et al., 2011; Sporns & Betzel, 2016), little is known about the modular structure of whole-brain networks at the mesoscale (Bruno et al., 2015; Lee et al., 2016; Vanni, Chan, Balbi, Silasi, & Murphy, 2017). Here, we leverage recent advances in modularity maximization, a data-driven method for uncovering a network’s modules, to uncover the hierarchical modular structure of spontaneous FC (Jeub, Sporns, & Fortunato, 2018; see **Materials and Methods** for more details).

**Figure 2.**
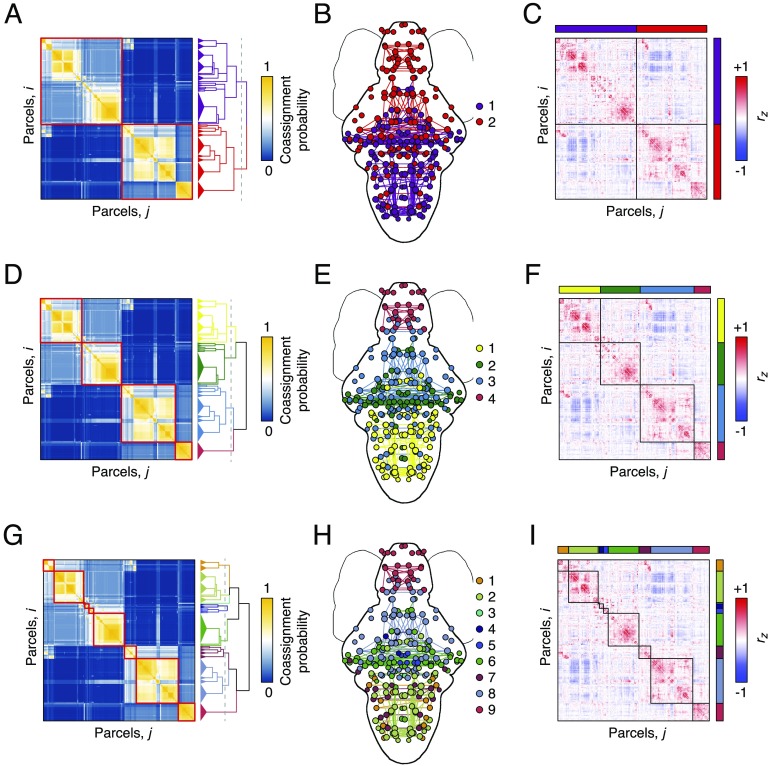
Hierarchical and multiresolution modular structure. (A) Coassignment matrix with hierarchy cut to highlight a two-module division (red blocks). (B) Modules plotted in anatomical space. Nodes are colored according to their module assignment, and within-module connections are colored similarly. (C) Correlation matrix ordered and blocked to highlight modular structure. Panels D–F and G–I show similar figures but with the number of modules equal to four and nine, respectively.

Modularity maximization returns hierarchically related partitions of nodes into modules, where the hierarchical levels are determined using a statistical criterion (Jeub et al., 2018). We found evidence of a hierarchy comprising 24 distinct levels, with the number of modules at any level ranging from 2 to 25 (Figure 2A). For the sake of brevity, we show partitions of nodes into *c* = 2 (Figure 2A–C), 4 (Figure 2D–F), and 9 (Figure 2G–I) modules (see Supporting Information Figure S4b–d for side projections of the same modules). We note that the quality of detected partitions, as indexed by the modularity index, *Q* (Newman & Girvan, 2004), was significantly greater in the empirical networks compared with the partitions of networks estimated from phase-randomized surrogate time series (*p* < 0.05, Bonferroni-corrected; see Supporting Information Figure S3).

Many of the modules recapitulate known functional and anatomical divisions of zebrafish. For instance, the red module (labeled 4 in Figure 2E and 9 in Figure 2H) overlaps closely the telencephalnon. Interestingly, this module isolates itself early within the hierarchy, and exhibits no clear subdivisions at deeper levels. Similarly, module 2 in Figure 2E and modules 4, 5, 6, and 8 in Figure 2H depict coarse and fine approximations of the mesencephalon. Unlike the telencephalic cluster, we identify multiple mesencephalic subdivisions, suggesting that these areas have the capacity to subtend varying levels of functional specialization.

Collectively, these findings suggest that spontaneous FC is organized hierarchically into modules that exhibit clear mappings to known neuroanatomy. These findings are in close agreement with analogous investigations of whole-brain networks at the macroscale (Meunier et al., 2009) and suggest that modular organization may be a unifying principle by which brain networks at all scales are structured (Taylor, Wang, & Kaiser, 2017).

### Hub Organization of Spontaneous FC

In the previous section we presented evidence that zebrafish spontaneous FC exhibits hierarchical modular structure. While segregated modules may be useful for the development of specialized brain function, complex behavior also requires network features that support the integration of information across different modules (Cohen & D’Esposito, 2016; Deco, Tononi, Boly, & Kringelbach, 2015; Sporns, 2013). One class of network feature that supports precisely this type of processing is hubs, nodes whose connections straddle the boundaries of modules. Here, we identify hubs in spontaneous FC using the network measure *participation coefficient* (Guimera & Amaral, 2005).

**Figure 3.**
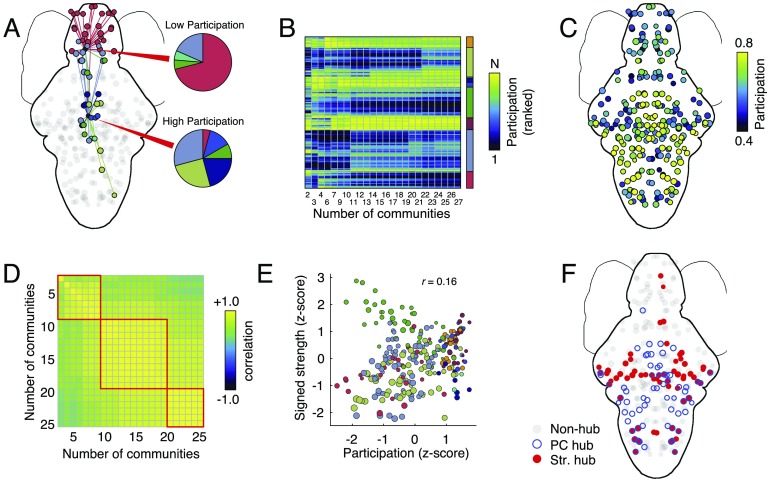
Participation coefficient and hub classification. (A) Schematic illustration of low- and high-participation nodes. Connections made by nodes with low participation (nonhubs) are concentrated within those nodes’ modules; connections made by nodes with high participation (hubs) are distributed across many modules. (B) Mean participation coefficient (ranked) as a function of the number of detected modules. (C) Average participation coefficient when the number of modules ranges from 2 to 25. (D) Correlation of brain-wide participation coefficient maps for different numbers of modules. While the changes in participation coefficient maps are subtle, there nonetheless appear to be three “regimes” (based on visual inspection) corresponding to distinct patterns of participation. (E) Participation coefficient and signed strength, z-scored and plotted against one another. Both measures serve as indices of “hubness” but are, on average, only weakly correlated. (F) Nodes in the top 25th percentile by participation and absolute strength. On average, there is little overlap.

Participation coefficient measures the uniformity with which a node’s connections are distributed across modules; values close to 1 or 0 indicate nodes that connect to many different modules or are concentrated within a small number of modules, respectively. We illustrate this concept schematically in Figure 3A, where we show examples of two nodes—one with low (top) and another with high (bottom) participation. We calculated each node’s participation coefficient at every hierarchical level and averaged these values over the entire hierarchy. In Figure 3B we show these coefficients after a rank transformation. On average, we find marked heterogeneity in the spatial locations of high-participation hubs, with the greatest concentration appearing within the rhombencephalon (Figure 3C). Though brain-wide patterns of participation coefficient are largely stable as we vary the number of modules, we nonetheless observe subtle scale dependencies (Figure 3D), suggesting that small subsets of nodes may be uniquely configured to act as hubs at one particular scale but less so at another.

To better contextualize these findings, we compared nodes’ participation coefficients with their absolute strength (total weight of functional connections), another measure sometimes used for identifying hubs (Figure 3E). In general, we find that these two metrics are only weakly correlated (*r* = 0.16; *p* < 0.05), that is, the most hub-like nodes according to participation coefficient and strength are not necessarily overlapping. Indeed, when we examine the top 25% of hubs according to each measure, we find little overlap between the two hub measures (Figure 3F).

These findings, combined with those from the previous section, suggest that spontaneous FC at the mesoscale strikes a precarious balance between features that promote segregated (i.e., local) brain function and those that promote integrative (i.e., global) brain function. These findings mirror those reported in macroscale networks (Cohen & D’Esposito, 2016), where the expression of hub areas has been linked to both genetics and cognitive performance (Bertolero, Yeo, Bassett, & D’Esposito, 2018). An important caveat here is that, typically, a node’s “hubness” is defined based on how it is situated with respect to a network’s modules (participation) *and* its capacity to exert influence over the network (degree/strength; Guimera & Amaral, 2005). Here, we study fully weighted and signed networks in which nodes have uniform degree and both positive and negative strengths (both of which are biased by community size; Power et al., 2013). Rather than risk misinterpreting a node’s capacity for influence, we focus entirely on the participation measure.

### Correspondence of Spontaneous and Stimulus-Evoked FC

In the previous sections, we focused on organizing principles of spontaneous (i.e., stimulus-free) FC. Spontaneous FC represents an intrinsic, baseline state in which functional connections are shaped primarily by an underlying network of physical pathways, projections, and fiber tracts rather than the functional demands of a task or stimulus (Goñi et al., 2014; Honey et al., 2009; Lin, Okun, Carandini, & Harris, 2015; Park & Friston, 2013). Nonetheless, nervous systems must be flexible and capable of reconfiguring in order meet those demands should they arise (Betzel, Bertolero, & Bassett, 2018; Cole et al., 2014). What features characterize stimulus-induced reconfiguration? Is it a wholesale reorganization of the network? Is it restricted to a small subset of connections or nodes? In this section, we explore the effect of different stimuli on FC organization.

To study stimulus-evoked changes in functional network organization, we estimated FC during the presentation of different visual stimuli. In addition to spontaneous activity, we considered phototaxis (PT), optomotor response (OMR), looming response (Looming), and dark-flash response (DF; see **Materials and Methods** for more details).

**Figure 4.**
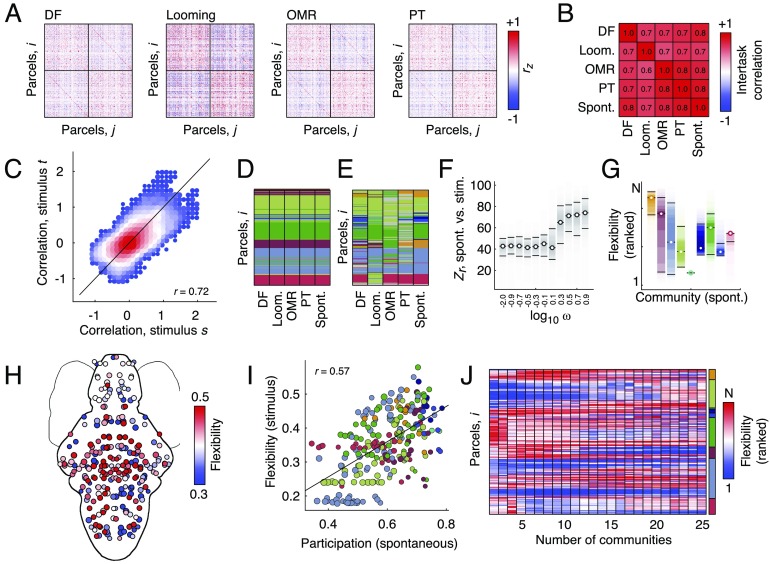
Comparing spontaneous and stimulus-evoked network architecture. (A) Functional connectivity matrices for four different stimulus conditions: dark-flash (DF), looming, optomotor response (OMR), and phototaxis (PT). (B) Upper triangle correlation for four stimulus conditions + spontaneous. (C) Scatterplot of all stimulus conditions plotted against one another. Size and color of each point indicate density of points at that location. Examples of (D) low-flexibility and (E) high-flexibility modules. In D and E, modules have been recolored to match the nine-module partition detected using the spontaneous network alone. Nodes with gray colors have module assignments that cannot be easily mapped to one of the nine modules. (F) Similarity (z-score of the Rand index) of stimulus-evoked and spontaneous modular structure as a function of the interlayer coupling parameter, *ω*. When *ω* is small, detected modules reflect specific stimuli; when *ω* is larger, detected modules are more similar to spontaneous modular structure. (G) Node-level flexibility grouped according to modules. Note: high levels of heterogeneity across modules. (H) Node-level flexibility plotted in anatomical space. (I) Correlation of stimulus-evoked flexibility and participation coefficient estimated from spontaneous network data alone (J) Variation of flexibility patterns as the number of detected modules changes.

First we estimated a FC matrix separately for each stimulus condition (Figure 4A). To assess the similarity of these matrices, we extracted their upper triangle elements, computed pairwise interstimulus correlations (Figure 4B), and also generated a scatterplot of these elements against one another (Figure 4C). Overall, we found that the stimulus-evoked connectivity matrices were highly similar (mean correlation of *r* = 0.72; *p* < 0.05, Bonferroni-corrected). Additionally, we statistically assessed the correspondence of spontaneous and stimulus-evoked correlation structure using the Mantel test (Mantel, 1967). For all combinations, we found that the correspondence was statistically significant (*p* < 0.05, Bonferroni-corrected; see Supporting Information Figure S9). These results support the hypothesis that the differences between spontaneous and stimulus-evoked connectivity is characterized by subtle shifts in connection weights and not by a wholesale reorganization of FC. In the next section, we explore these subtleties in greater detail.

### Hub Nodes Reconfigure in Response to Stimuli

In the previous section we found that stimulus-evoked and spontaneous FC were highly correlated with one another across different stimulus conditions. In this section, we tease apart those differences. To address this question, we adapted a multilayer modularity maximization procedure (see **Materials and Methods** for more details; Mucha, Richardson, Macon, Porter, & Onnela, 2010), which has been used to study time-varying FC (Bassett et al., 2011) and interindividual differences at the macroscale (Betzel, Bertolero, Gordon, et al., 2018). This procedure entails treating each spontaneous and stimulus-evoked FC matrix as a “layer” in a multilayer network ensemble. Layers are coupled to one another and the entire ensemble used as input for modularity maximization, thereby estimating modules in all layers simultaneously. As a result, this procedure allows us to effortlessly map modules across stimulus conditions and to identify flexible and inflexible nodes, that is, those whose module assignments are consistently variable versus those that are stable.

The multilayer modularity maximization framework includes an additional free parameter, *ω*, that can be tuned to different values so that the detected modules emphasize either the unique modular structure for each stimulus condition or the shared modular structure across conditions. When *ω* is large (emphasizing common modular structure), the detected multilayer modules are highly similar to those detected in the previous section using the spontaneous networks, alone (Figure 4D). This result is expected given the high correlation between stimulus-evoked and spontaneous FC. Here, we show as an example the module assignments that best match the nine-module partition shown in Figure 2G. When *ω* is set to smaller values, however, the algorithm detects modules that are uniquely suited to each layer and therefore more variable (Figure 4E). We see this more clearly when we compare stimulus-evoked and spontaneous modular structure as a function of *ω* (Figure 4F). The average similarity of stimulus-evoked and spontaneous modular structure, as measured by the z-score of the Rand index (Traud, Kelsic, Mucha, & Porter, 2011), increases monotonically as a function of *ω*.

Because the multilayer modularity maximization framework preserves module labels across layers, it facilitates straightforward comparisons of modules from one stimulus condition with modules from another. This allows us to calculate each node’s “flexibility” (Bassett et al., 2011, 2013), a measure of its network variability across stimuli (see **Materials and Methods**). Intuitively, nodes with high flexibility are those that frequently switch module allegiance in response to a stimulus, whereas low-flexibility nodes are invariant and serve as stable anchors of network organization across different conditions. Here, we calculated the average flexibility for each node across all sets of multilayer modules for which the mean number of modules was between 2 and 25. Interestingly, we found that flexible nodes were not uniformly distributed across modules, but exhibited high levels of heterogeneity (Figure 4F, G). Perhaps most surprising, when we compared participation coefficient (estimated from spontaneous FC alone) with flexibility, we found a strong positive correlation (*r* = 0.57; *p* < 0.05), indicating that spontaneous network structure may play a role in shaping network responses to stimuli.

Collectively, these findings suggest that spontaneous, intrinsic network architecture represents a powerful constraint on FC during stimulus-evoked conditions, in terms of both connectivity patterns as well as modular structure. More specifically, we find a common, stable modular core around which a flexible periphery of nodes reconfigure their connections as they adapt and respond to ongoing stimuli (Oram, 2010). More importantly, the nodes that appear most capable of reconfiguration under task conditions are the same nodes that occupy positions of influence with respect to the spontaneous modular structure. These findings further validate observations made at the macroscale, wherein high participation areas overlap with polyfunctional association cortex (Cole et al., 2013; Power et al., 2013; Zanto & Gazzaley, 2013). We note, however, that while the link between participation at rest and flexibility across tasks is not without precedent, for example, control networks having high task flexibility (Cole et al., 2013) and participation (Power et al., 2013), this relationship is not a mathematical necessity. The observations used to estimate spontaneous and task FC were independent from one another. Accordingly, there is no mathematical reason to expect that participation and flexibility should be related to one another.

### Stability of Network Architecture Across Individuals

**Figure 5.**
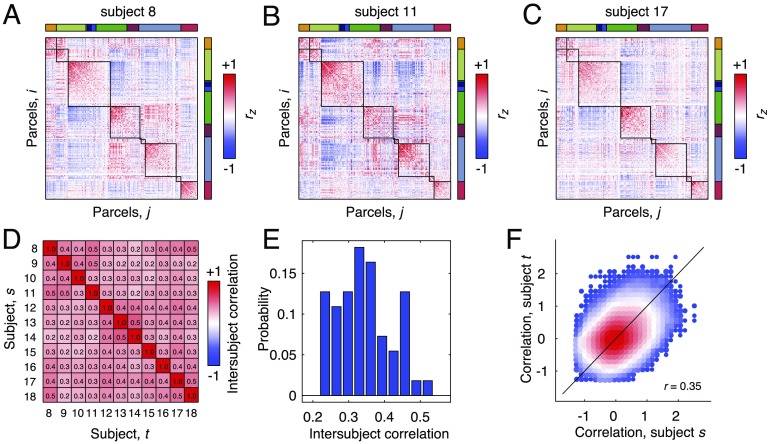
Intersubject similarity of spontaneous FC. Panels A, B, and C depict single-subject spontaneous FC matrices ordered according to the nine-module partition shown in Figure 2G. (D) Correlation (similarity) matrix of upper triangle elements in subject-specific spontaneous FC matrices. (E) Distribution of elements in upper triangle of correlation matrix shown in panel D. (F) Scatterplot of functional connections for subjects *s* and *t*.

In the previous sections, we demonstrated that spontaneous FC is organized by geometry, exhibits hierarchical modules and hubs, and constrains stimulus-evoked FC. In this final section, we conclude by showing that the network structure of spontaneous FC appears to be conserved and similar across individual subjects. To compare network structure across subjects, we estimated spontaneous FC networks separately for subjects 8–18. We note that the decision to focus on this subset of subjects was motivated practically; at the time of data accession, only data from fish 8–18 were fully annotated. For subjects 1–7, we were unable to determine experimental state, such as spontaneous versus stimulus, from the data descriptors. Nodes and connections were defined exactly as before. In Figure 5A–C we show example FC matrices for subjects 8, 11, and 17.

Upon visual inspection alone, the similarity of these matrices is apparent. Nonetheless, we quantified their similarity precisely by computing the correlation of their upper triangle elements with one another (Figure 5D). We found that the mean correlation was *r* = 0.35 ± 0.08, indicating that while the correspondence of network architecture across subjects was imperfect, it was nonetheless robustly similar. We visualize these findings in two different ways: In Figure 5E we plot the distribution of intersubject similarity and in Figure 5F we show the two-dimensional histogram of connections plotted against one another. We also report a robust correspondence of spontaneous and task-evoked connectivity (mean correlation of rest to all tasks of *r* = 0.45 ± 0.10) and evidence that, averaged across tasks, subjects are more similar to themselves than to other subjects (mean self-similarity of *r* = 0.46 ± 0.08 and intersubject similarity of *r* = 0.34 ± 0.04; Finn et al., 2015; Supporting Information Figure S8).

In summary, these findings indicate that network structure of spontaneous FC is reproducible and conserved across individuals. Further, they suggest the presence of a neuro-functional blueprint shared across individuals. These findings contribute to the growing literature on uncovering sources of intersubject variability in FC (Gordon et al., 2017; Seghier & Price, 2018) and extend this research domain from the macro- to the mesoscale.

## DISCUSSION

Nervous systems are fundamentally complex networks of interacting neurons, neuronal populations, and brain areas. Current research into the structure of these networks has begun to reveal their basic organizing principles. Despite this, little is known about the architecture of biological neural networks at the mesoscale (at the level of groups or clusters of individual neurons). Here, we use network science methods to investigate the organization of mesoscale networks in larval zebrafish during spontaneous and stimulus conditions. Our analyses reveal the effect of geometry on network structure, a modular architecture and the hubs that span module boundaries, and the constraint of intrinsic network structure on stimulus-evoked connectivity. These features are analogous to those observed in whole-brain networks constructed at the macroscale; our findings and methods therefore serve as conceptual bridges, linking investigations of nervous system structure and function across organizational scales. The work presented here serves as a methodological blueprint for future mesoscale network analyses and highlights several outstanding neuroscientific questions to be addressed with additional experiments and modeling.

### Bridging Scales

Nervous systems exhibit meaningful organization and behavior across a wide range of spatial scales—from the level of cells and molecules up to brain areas (Betzel & Bassett, 2017b). However, different spatial scales are imaged using different technologies, resulting in data that are often modeled and analyzed using different statistical and mathematical methods. These distinctions in how spatial scales are researched can give rise to sometimes competing or contradictory accounts of nervous system organization and behavior; this is especially true when the neural phenomenon being studied is not clearly restricted to a single scale. Accordingly, there is a growing need for theoretical frameworks capable of explaining and modeling observations simultaneously at many scales.

In the present work, we use network science methodology to model and characterize whole-brain mesoscale networks. Though these methods have been widely used at the macroscale (Bullmore & Sporns, 2009; He & Evans, 2010; Rubinov & Sporns, 2010), they are only beginning to be applied to mesoscale functional datasets and have been restricted to network reconstructions at the population level (Bruno et al., 2015; Faber et al., 2018; Muldoon, Soltesz, & Cossart, 2013; Orlandi et al., 2013; Yamamoto et al., 2018) or at the level of entire brains but without cellular resolution (Barson et al., 2018; Lake et al., 2018; Vanni et al., 2017). In extending the network approach to the whole-brain level and for networks reconstructed from single-cell observations, we provide a powerful demonstration of the utility of network science for gaining insight into nervous system architecture. Moreover, our work showcases network science as a framework with the ability to form bridges across spatial scales and, potentially, help reconcile disparate findings (Bassett & Sporns, 2017).

### Universal Organizational Principles

One of the goals of network neuroscience is not simply to *describe* a network, but to identify the principles by which nervous systems are organized (Bassett & Sporns, 2017). In other words, what are the rules by which a network’s structure is shaped (Betzel & Bassett, 2017a; Ercsey-Ravasz et al., 2013; Goulas, Betzel, & Hilgetag, 2018; Vértes et al., 2012) and are those rules universally true or do they apply only to particular organism and scale (van den Heuvel et al., 2016)? This goal is challenging to address, as most research that constitutes network neuroscience has focused on a single scale (macro) for a single organism (human), limiting the possibility of discovering scale- and species-invariant organizing principles.

Here, because of recent advances in cellular-level imaging (Ahrens et al., 2012; Vladimirov et al., 2014), we constructed whole-brain mesoscale functional networks and found, surprisingly, that these networks express analogous features as macroscale networks, suggesting that similar rules and principles are responsible for their organization. These features include connection weights with a strong geometric dependence, a balance between segregated and integrated brain function, and subtle stimulus-induced reconfigurations of network architecture. These findings indicate that functional networks reconstructed at the mesoscale may be organized according to an analogous set of rules and principles, suggesting that these rules may be approximately scale-invariant. Moreover, these findings also suggest that, despite increases in dimensionality at the mesoscale, network organization can be succinctly described by a low-dimensional set of principles.

### Future Directions

There are several ways that the work presented here could be extended in the future. First, although the network science methods we used provided novel insight into network structure at the mesoscale, those methods are relatively established in the network neuroscience literature (Guimera & Amaral, 2005; Newman & Girvan, 2004; Rubinov & Sporns, 2010). Recently, increasingly novel methods have been developed that assay more nuanced aspects of network structure; in the long term, it would be useful to test whether these new (but more complex) approaches based on algebraic topology (Saggar et al., 2018), control theory (Gu et al., 2015), blockmodeling (Betzel, Medaglia, & Bassett, 2018), and graph signal processing (W. Huang et al., 2018) can give novel insights into mesoscale network structure and function. In the near term, a potentially more fruitful approach could involve estimating time-varying FC to track fluctuations in network structure over short timescales and thereby gain insight into network dynamics (Hutchison et al., 2013).

Second, though networks analyzed here were reconstructed from single-cell recordings, we aggregated cells into parcels that we regarded as nodes. Though the parcels were defined to be both spatially and functionally homogeneous, each parcel-averaged fluorescence trace likely does not perfectly explain the variance of traces for every cell assigned to that parcel. In future work, it is critical to explore the effect of alternative parcellations on network statistics (Wang et al., 2009). Additionally, it would be useful to explore parcellation-free approaches, wherein connections are estimated between individual cells (though this approach will likely scale poorly as the number of recorded cells increases; Kim et al., 2016).

Additionally, our work sets the stage for future studies to form much stronger bridges between observation and theory. In network neuroscience, specifically, there is no shortage of theories concerning the functional roles of particular classes of network structures. For example, it remains unclear exactly what role connections play in shaping communication patterns between neural elements, and whether this process is selective, for example, biased towards the utilization of specific pathways, or unguided, as in a random walk or diffusion process (Avena-Koenigsberger, Misic, & Sporns, 2018). Testing hypotheses like these is challenging, largely because the experimental perturbations necessary, such as ablating cells or pathways, are invasive and because most macroscale networks are constructed noninvasively in human subjects, where invasive perturbations are, in general, not possible (with some notable exceptions; Solomon et al., 2018). Extending network analysis to the mesoscale and to model organisms like zebrafish makes it possible to leverage focused perturbations (Yamamoto et al., 2018) and novel neuroscientific techniques (Vladimirov et al., 2018), to confront, test, and disambiguate network-level hypotheses about brain function.

Another important direction for future work involves the comparison of the functional imaging data analyzed in this study and made available via (X. Chen et al., 2018), with additional biological data collected and disseminated as part of other neuroinformatics studies. For instance, paralleling structure-function work carried out at the macroscale (Betzel et al., 2019), future studies could establish an anatomical basis for correlated activity at the mesoscale and cellular levels through detailed comparisons against physical connections (Kunst et al., 2019). Similarly, and by virtue of working with model organisms, the genetic mechanisms that underlie patterns of correlated activity can be probed in much greater detail than is possible at the macroscale by, for example, taking advantage of publicly available gene expression atlases (Sprague, Doerry, Douglas, & Westerfield, 2001).

While this work includes analysis of functional brain networks reconstructed from cellular-level recordings, to facilitate intersubject comparisons it was nonetheless necessary to cluster cells into a set of parcels. This aggregation step resulted in a consistent set of network nodes across all individuals. Future work should explore granular approaches for analyzing these networks that do not sacrifice the single-cell resolution of these data, such as by embedding subject-level networks in the same space (Simas, Chavez, Rodriguez, & Diaz-Guilera, 2015). Though this work helps clarify the organizational features and constraints of biological neural networks at the mesoscale, future studies of single-cell organization will provide even greater clarity (Ponce-Alvarez, Jouary, Privat, Deco, & Sumbre, 2018).

### Limitations

Despite contributing to our understanding of the organizing principles underpinning mesoscale biological neural networks, this study has a number of important limitations. First, it is critical to note that our measure of connectivity is a linear correlation. Though this particular measure has been used extensively to model functional connectivity of slowly evolving fMRI BOLD data (David, Cosmelli, & Friston, 2004) and performs well in recovering underlying network structure when applied to synthetic time series data (Smith et al., 2011), it is not a direct measure of structural connections (axonal projections), it is not equivalent to the coupling matrix in a dynamical systems model, does not indicate directedness of a connection, and is in no way a measure of causality or mechanism. Consequently, the results of all analyses are descriptive in nature. In future work, perturbational experiments and novel network reconstruction techniques will help clarify the causal role of network organization (L. Huang et al., 2018; Lansdell & Kording, 2018).

Second, we infer connection weights based on zero-lag correlations of calcium fluorescence, which serves as an indirect measurement of a neuron’s activity. This indirectness, coupled with lack of temporal precision (two volumes acquired per second), opens to the possibility that reconstructed networks are not fully recapitulating the “true” correlation structure of neurons’ activities. Advances in electrophysiological methods that enable recording from a large number of neurons (Jun et al., 2017) and optical techniques for accelerating acquisition times (Sadovsky et al., 2011) will prove useful in addressing these and related issues in future work.

Finally, another limitation concerns the methodology used for parcel (node) definition. Here, we combined data from all subjects and all stimulus conditions to generate spatially contiguous and functionally homogeneous parcels, which is common in the large-scale imaging literature (Eickhoff, Yeo, & Genon, 2018). In principle, these parcels would be validated in an independently acquired dataset to ensure their robustness and reduce the risk of overfitting. However, because of the uniqueness of the present dataset, finding a second comparable and independent dataset was not possible. Relatedly, parcels were defined to be similar across subjects and stimulus conditions. However, recent large-scale imaging work has shown that parcel boundaries fluctuate with both individuals and task (Salehi et al., 2019). As similar datasets become available in the future, the validity and robustness of parcels should be investigated further using cross-validation procedures. Similarly, future work should explore alternative parcellations that may better reflect subject-level and stimulus-specific network organization.

## MATERIALS AND METHODS

### Data Acquisition

Activity from a majority of neurons was recorded in larval zebrafish using light-sheet microscopy (Vladimirov et al., 2014). Activity was recorded under spontaneous conditions and also while subjects were presented with a series of visual stimuli. As reported in X. Chen et al. (2018), calcium imaging data were recorded at ≈ 2 volumes/second for ≈ 50 min (or ≈ 6,800 volumes). As in Vladimirov et al. (2014), the calcium indicator GCaMP6f (T.-W. Chen et al., 2013), was expressed pan-neuronally and fused to cell nuclei, allowing for the automatic segmentation of cells (Kawashima, Zwart, Yang, Mensh, & Ahrens, 2016). The result was continuous fluorescence traces for ≈ 80,000 cells per subject. As noted in X. Chen et al. (2018), this number accounts for the majority of neurons in the brain, excluding extremely ventral areas. For complete details of data acquisition, see X. Chen et al. (2018).

### Spontaneous Activity and Visual Stimuli

Most of our analyses focused on networks reconstructed from spontaneous activity, that is, in the absence of any visual stimuli. During this condition, which lasted on average 419.0 ± 131.4 s, the fish engage in fictive swimming through a series of alternating left- and right-turn sequences (X. Chen et al., 2018; Dunn et al., 2016).

In addition to spontaneous activity, subjects were imaged concurrent with the presentation of visual stimuli, the precise timing of which varied from subject to subject (see X. Chen et al., 2018, for complete details). Briefly, these stimuli included the following:

1.  *Phototaxis*: Subjects were presented with half-field black and white visual stimuli on either side. Presentations were followed by a whole-field black stimulus. On average, the half-field black and white stimuli were presented for 17.9 ± 5.2 s, interspersed by the whole-field black stimulus, lasting for 7.04 ± 3.6 seconds. This repeated motif of whole-field black to half-field white to whole-field black to half-field black lasted for 759.7 ± 331.7 s.
2.  *Optomotor response*: Whole-field stripes moving in different directions. Here, some subjects were presented with three different movement directions and others with four, the precise timing of which was variable. Presentation lasted 8 ± 3.9 s, between which were blank stimuli lasting approximately 9.6 ± 4.3 s. The total duration of this stimulus was, on average, 631.5 ± 303.1 s.
3.  *Looming response*: Expanding discs on either left or right side interspersed by long periods of no stimulus. The disc periods alternated and lasted, on average, 3.6 ± 1.3 s, while the nonstimulus periods lasted 18.1 ± 6.6 s. This stimulus lasted 350.0 ± 158.7 s.
4.  *Dark-flash response*: Sudden darkening of environment lasting, on average, 15.2 ± 6.7 s, followed by lightening, lasting almost an equal amount of time, 16.1 ± 7.2 s. This stimulus lasted, on average, 335.2 ± 109.3 s.

### Node Definition

Rather than analyze networks where nodes correspond to individual cells, we focused on networks where the nodes represented clusters of cells, effectively reducing computational burden and facilitating straightforward interpretation. We developed a data-driven approach to assign cells to clusters that possessed three distinct properties: (a) spatial contiguity, (b) functional homogeneity, and (c) intersubject consistency.

To generate clusters with this set of properties, we designed a multistage clustering algorithm. First, we aggregated normalized spatial coordinates of cells in the left hemisphere across all subjects and clustered them using k-means into *k* = 2,500 contiguous spatial clusters (distance metric = Euclidean; number of replicates = 10). This aggregation step was possible because cells had previously been aligned across subjects (X. Chen et al., 2018). We then mirrored the centroids about the midline and assigned cells in the right hemisphere to the nearest spatial cluster centroid (5,000 hemispherically symmetric clusters; mean spatial cluster size of 280 ± 73 cells). Because this procedure defines spatial clusters based on cells’ spatial locations, the resulting spatial clusters were also spatially contiguous. Moreover, because spatial clusters were defined using aggregated coordinates, spatial clusters contained cells from multiple subjects. Of the 5,000 spatial clusters, 82.6% contained cells from all subjects and 92.5% contained cells from at least 75% of subjects.

Next, we concatenated spontaneous and stimulus-evoked activity and regressed the global signal from each cell’s fluorescence trace (Power et al., 2014). This procedure ensured that any observed fluctuations in fluorescence were not driven by changes in baseline fluorescence. (Note: We also carried out select sets of analyses on data in which the global signal regression step was omitted and found generally similar results, but with baseline correlation increased and more clear monotonic decay of connection weight as a function of distance; see Supporting Information Figure S5.) Then for each spatial cluster and each subject, we extracted a cluster-averaged fluorescence trace, and computed pairwise correlations for all spatial clusters. This correlation structure reflects both spontaneous and stimulus-evoked activity and effectively helps mitigate the possibility that subsequent clusters are overfit and biased towards the correlation structure of one condition or another. We repeated this algorithm separately for both each hemisphere before averaging over subjects *and* hemispheres, resulting in a single 2,500 × 2,500 matrix of group- and hemisphere-averaged correlations (we were able to average over hemispheres because spatial clusters were defined to be hemispherically symmetric and so each spatial cluster had a homotopic partner). Next, we used this matrix to identify functional clusters with high average functional homogeneity by clustering its rows using k-means. We asked the algorithm to generate *k* = 100 functional clusters (distance = correlation; number of replicates = 10). However, the resulting functional clusters were, in general, no longer spatially contiguous (i.e., functional clusters could include spatial clusters whose fluorescence traces were similar, but not necessarily proximal to one another). Accordingly, we extracted all spatial clusters assigned to each functional cluster and further divided them according to their spatial coordinates until the maximum diameter was <200 pixels and each spatial cluster in a functional cluster was no fewer than 60 pixels from any other spatial cluster assigned to the same functional cluster. In the end, this procedure resulted in *N* = 256 *parcels*, each of which represented a node in our network.

We note that this clustering procedure necessarily sacrifices some of the resolution at which the original data were recorded. That is, by consolidating single cells into clusters, the unique identities of those cells (and any information carried by fluctuations in their activity over time) is lost. However, in doing so, we generate parcels whose identities are preserved across individuals, thereby facilitating intersubject comparisons that would have, otherwise, been difficult to carry out.

### Network Construction

To construct networks, we mapped the *N* = 256 nodes defined in the previous section back to individual subjects and to single cells. For each subject and for each node, we extracted the average fluorescence trace of its constituent cells and computed the pairwise correlation for all pairs of nodes. All correlation coefficients were subsequently Fisher transformed. We also confirmed that the observed correlation patterns were not driven by low-level statistical properties of the fluorescence traces. For each subject, we generated 1,000 phase-randomized surrogates in which we calculated the discrete Fourier transform of each fluorescence trace, added random phase offsets, and took the inverse transform (Theiler, Eubank, Longtin, Galdrikian, & Farmer, 1992). We then estimated the correlation structure of the resulting surrogate time series (as a Spearman correlation). We found that the surrogate time series generate wildly different correlation patterns compared with the observed network (average mean similarity across subjects of *ρ* = −3.6 × 10^−4^ ± 5.9 × 10^−4^). This strongly suggests that the correlation structure estimated from the original time series is not driven by low-level statistics of the calcium fluorescence traces.

### Multiresolution Modularity Maximization

Modularity maximization is a flexible, data-driven method for partitioning networks into subnetworks called “communities” or “modules” (Newman & Girvan, 2004). It operates according to a simple principle—compare observed connections against what would be expected under a null connectivity model. Modules, then, are groups of nodes whose observed internal density of connections is maximally greater than expected. This intuition is quantified using the modularity heuristic, *Q*, defined as the following:

$$Q(\gamma ,\{g_{i}\})=\underset{ij}{\sum }[A_{ij}-\gamma \cdot P_{ij}]\delta (g_{i},g_{j}).$$ (1)

Here, *A*_*ij*_ and *P*_*ij*_ are the observed and expected connection weights between nodes *i* and *j*; *γ* is the structural resolution parameter; *g*_*i*_ ∈ {1, …, *C*} is the module assignment of node *i*; and *δ*(*x*, *y*) is the Kronecker delta function, where *δ*(*x*, *y*) = 1 when *x* = *y* and is *δ*(*x*, *y*) = 0, otherwise. Effectively, this function calculates the total weight of within-module connections less that of the null model. Partitions that correspond to larger values of *Q* are considered to be, objectively, of higher quality. Here, we used a uniform null connectivity model, that is, *P*_*ij*_ = 1, which helps mitigate issues related to the resolution limit (Traag, Van Dooren, & Nesterov, 2011) and is appropriate for use with correlation matrices (Bazzi et al., 2016). We note that under this null model the expected magnitude of correlation is controlled by *γ* parameter.

#### Hierarchical modules.

Here, we used two variants of modularity maximization to detect modules in zebrafish functional networks. The first seeks to relate coarse (large) and fine (small) modules to one another through a hierarchy (Jeub et al., 2018; http://github.com/LJeub/HierarchicalConsensus). This procedure takes advantage of the resolution parameter, *γ*, which can be tuned to detect modules of different sizes. Briefly, we sampled 10,000 partitions at various values of *γ*, which results in modules of different sizes. We then summarized those modules using a coassignment matrix whose elements indicated the fraction of all samples in which pairs of nodes were assigned to the same module. To generate a hierarchy, this coassignment matrix was recursively clustered, with larger modules divided into smaller modules according to a statistical criterion. This procedure resulted is a series of hierarchically related modules.

#### Multilayer modules.

We also used a second variant of modularity maximization, in which the *Q* heuristic is extended to “multilayer” networks (Mucha et al., 2010). Here, layers represented connectivity matrices estimated under different stimulus conditions. For each layer, *s*, we calculated its corresponding modularity matrix, *B*_*s*_ = *A*_*s*_ − *γ* ⋅ *P*_*s*_. Node *i* in layer *s* was then linked to itself in all other layers by an interlayer connection of weight *ω*. Next, modules were detected for all layers simultaneously by submitting the entire multilayer ensemble—all layer-specific modularity matrices and interlayer connections—to the modularity maximization algorithm (see Supporting Information Figure S6 for a schematic). The corresponding *Q* heuristic can be written as follows:

$$Q(\gamma ,\omega ,\{g_{is}\})=\underset{ijsr}{\sum }[B_{ijs}]\delta (g_{is},g_{js})+\delta (i,j)\cdot \omega ]\delta (g_{is},g_{jr}).$$ (2)

This multilayer formulation has the distinct advantage over single-layer formulations, in that by detecting modules in all layers simultaneously, module labels are preserved across layers. That is, the observation of the same module label in layers *s* and *t* ≠ *s* indicates a recurrence of a module. This fact facilitates straightforward module comparisons and enables the computation of a multilayer flexibility measure, *f*_*i*_, whose value indicates the fraction of times that a node *i*’s module assignment differs across pairs of layers.

$$f_{i}=1-\frac{2}{T(T-1)}\underset{r,s>r}{\sum }\delta (g_{i,r},g_{i,s}).$$ (3)

In the context of the current study, high-flexibility nodes represent nodes whose community assignment varies from one stimulus condition to another. Low-flexibility nodes, on the other hand, refer to nodes whose community assignment is invariant across conditions.

#### Louvain algorithm.

For both the hierarchical and multilayer variants of modularity maximization, we used the generalized Louvain toolbox to optimize *Q* (Jutla, Jeub, & Mucha, 2011). In both cases, we sampled 10,000 values of *γ* within the range [−0.05,1.285]. The value of *γ* = −0.05 was determined by experimentation and represented the approximate smallest value of *γ* for which the network was partitioned into two or more communities. The second value of *γ* = 1.285 represented a theoretical upper limit, above which the optimal partition was always to assign each node to a singleton community. Therefore, this range bounds all “interesting” partitions—below which all nodes were assigned to one big community and above which all nodes were assigned to singleton communities. We sampled *γ* values within this range to approximate the distribution of connection weights in the network. Specifically, we sampled 10,000 times with replacement from the set of connection weights that fell within the acceptable range. We relied on a similar approach for sampling *γ* values when we used multilayer modularity maximization.

The *ω* parameter enters into modularity maximization only in the multilayer case and controls the heterogeneity of communities across layers (conditions). When *ω* is small, communities reflect organization unique to each layer; when *ω* is large, they reflect organization that is shared across layers. Here, we sampled *ω* from logarithmic distribution. That is, we first selected 10,000 exponents between the values of [−3, 1] and obtained *ω*s by raising the value of 10 to the power of each sample. Accordingly, the smallest and largest possible values of *ω* were 0.001 and 10.

As described in a recent paper (Betzel, Bertolero, Gordon, et al., 2018), we found that variation in *ω* effectively changed the baseline level of community variability (flexibility) but had little influence on the overall *pattern* of flexibility. So when we remove the effect of baseline differences in flexibility by rank transforming flexibility (as we do in Figure 4), the flexibility patterns over which we average tend to be homogeneous.

#### Comparing modular structure.

To compare two modular partitions, we used the z-score of the Rand index (Traud et al., 2011), a similarity measure that, intuitively, corrects the more common Rand index for the number and size of communities in the partitions. For two partitions, *X* and *Y*, we calculate their similarity as

$$Z_{r}(X,Y)=\frac{1}{\sigma_{w_{XY}}}w_{XY}-\frac{W_{X}W_{Y}}{W}.$$ (4)

Here, *W* is the total number of node pairs in the network, *W*_*X*_ and *W*_*Y*_ are the number of pairs in the same modules in partitions *X* and *Y*, respectively, *w*_*XY*_ is the number of pairs assigned to the same module in *both* *X* and *Y*, and *σ*_*w*_*XY*__ is the standard deviation of *w*_*XY*_. The value of *Z*_*r*_(*X*, *Y*) can be interpreted as how great, beyond chance, is the similarity of partitions *X* and *Y*.

### Defining Network Hubs

Hubs are considered nodes of disproportional importance to a network. We use three measures to identify hubs. The first measure is the flexibility metric. Nodes with high flexibility are those whose module affiliation changes across stimulus conditions and are therefore able to reconfigure in response to different stimuli or tasks.

The second metric used for hub classification is a node’s absolute strength—that is, the total weight of all of its connections:

$$s_{i}=\underset{j}{\sum }|A_{ij}|.$$ (5)

Intuitively, nodes with stronger connection weights, either positive or negative, may occupy positions of influence within the network.

Finally, we also defined hubs to be nodes with large participation coefficient (Guimera & Amaral, 2005). A participation coefficient measures how uniformly a node’s connections (positive, in this case) are distributed across modules. A node that makes positive connections to many different modules will have a high participation coefficient, while a node whose connections are restricted to a small number of modules will have a low participation coefficient. More specifically, participation is calculated as

$$p_{i}^{+}=1-\overset{N_{c}}{\underset{c=1}{\sum }}{\left(\frac{\kappa_{ic}^{+}}{k_{i}^{+}}\right)}^{2}.$$ (6)

Here, *N*_*c*_ is the number of modules, $\kappa_{ic}^{+}$ is the total weight of positive connections from node *i* to module *c*, and $k_{i}^{+}$ is the total weight of positive connections incident upon node *i*. Intuitively, nodes whose connections are distributed uniformly across modules have values of $p_{i}^{+}$ close to 1, while nodes whose connections are concentrated within a small number modules have values of $p_{i}^{+}$ closer to 0. Participation coefficient was computed using functions from the Brain Connectivity Toolbox (Rubinov & Sporns, 2010).

## ACKNOWLEGMENTS

RFB is grateful to M. Ahrens and X. Chen for sharing zebrafish imaging data. RFB thanks Olaf Sporns and Joshua Faskowitz for reading an early draft and providing invaluable feedback. RFB acknowledges support from that National Institute of Biomedical Imaging and Bioengineering (1R01EB029272-01). This research was supported by Indiana University Office of the Vice President for Research Emerging Area of Research Initiative, Learning: Brains, Machines and Children.

## DATA AVAILABILITY

All imaging data are available courtesy of M. Ahrens and X. Chen (https://github.com/xiuyechen/fishexplorer) (Jeub et al., 2018). All analysis scripts used in this manuscript are available from the authors upon request.

## SUPPORTING INFORMATION

Supporting information for this article is available at https://doi.org/10.1162/netn_a_00121.

## AUTHOR CONTRIBUTIONS

Richard Betzel: Conceptualization; Formal analysis; Investigation; Methodology; Software; Visualization; Writing - Original Draft; Writing - Review & Editing.

## FUNDING INFORMATION

Richard Betzel, National Institute of Biomedical Imaging and Bioengineering (US), Award ID: 1R01EB029272-01.

## TECHNICAL TERMS

Functional connectivity: Statistical associations between activity recorded at different locations in the brain.
Hubs: Nodes whose connections span modular boundaries, linking modules to one another.
Mesoscale: Spatial organization of cell-to-cell connectivity aggregated into parcels, regions, or areas.
Hierarchical and multiscale modules: Smaller modules nested within larger modules; may span multiple hierarchical levels.
Geometric organization: Dependence of functional connectivity on the spatial organization of the brain.
Modules: Cohesive groups of nodes that are segregated from the rest of the brain.
Flexibility: Nodes whose module assignment frequently changes (here, this change is across different stimulus conditions).
