# Supplementary material for: Organizing principles of whole-brain functional connectivity in zebrafish larvae
Source: Netw Neurosci. 2020 Mar 1;4(1):234–56. doi: 10.1162/netn_a_00121 (PMC7055648; doi:10.1162/netn_a_00121)
Supplement: Supplementary file 1 [file netn-04-234-s001.pdf]

Betzel, R. F. (2020). Supporting information for "Organizing principles of wholebrain functional connectivity in zebrafish." *Network Neuroscience*, *4*(1), 234–256.
https://doi.org/10.1162/netn_a_00121

Supplementary Figures: Organizing principles of functional networks in zebrafish larvae

Richard F. Betzel[1−4*]
[1] *Department of Psychological and Brain Sciences,*
[2] *Cognitive Science Program,* [3] *Program in Neuroscience,*
[4] *Network Science Institute, Indiana University, Bloomington, IN 47405*
(Dated: December 10, 2019)

* rbetzel @ indiana.edu

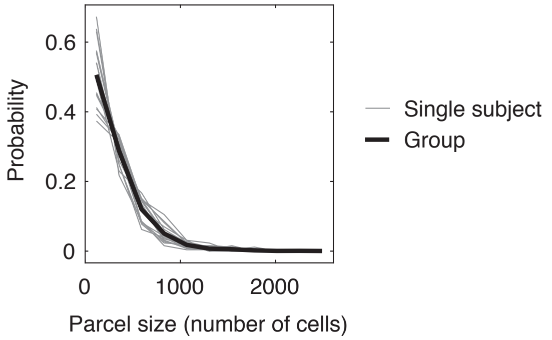

FIG. S1. **Number of cells per parcel.** Histogram of the number of cells in each of the $N = 256$ parcels for each subject. The gray lines represent individual subjects and the black line represents the pooled group average. On average, the interquartile range of cells per parcel was $[94, 416]$. The largest parcel observed in any subject, in terms of number of cells, included 2605 cells.

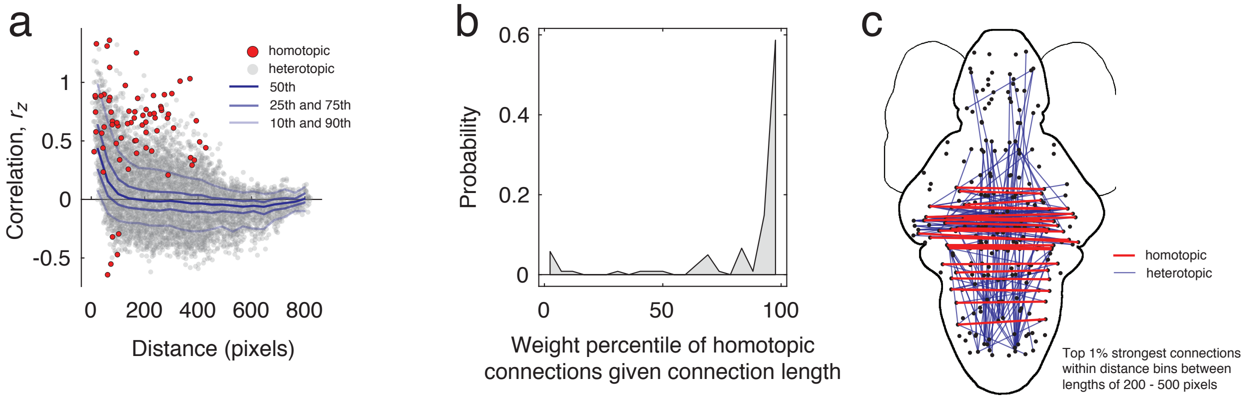

FIG. S2. **Analysis of homotopy and long-distance correlations.** (a) Scatterplot of edge weight *versus* distance with homotopic (red) and heterotopic (gray) connections labeled. We also include blue guidelines representing the median, 50th/75th, and 10th/90th percentiles across 21 distance bins. (b) For each homotopic connection, we calculated the fraction of all heterotopic connections of similar length but with weaker connection weight. (c) While homotopic connections were among the strongest connections in the network given their lengths, there were many heterotopic connections of similarly stronger-than-expected weight. Here, we show the top 1% of connections in distance bins from 200 - 500 pixels.

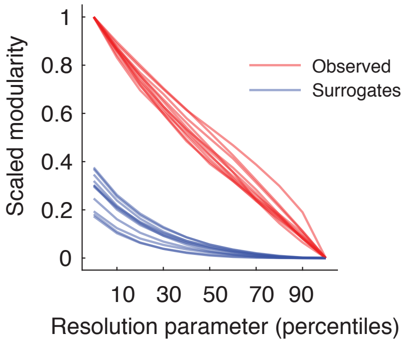

FIG. S3. **Comparing the modularity, Q, of observed and surrogate data.** We used modularity maximization with 10 repetitions of the Louvain algorithm to estimate the optimal partition at 11 different γ values and across all subjects. This procedure was carried out on networks estimated from the observed data, but also from networks estimated from phase-randomized surrogate time series (100 surrogates per subject). We compared the observed and surrogate modularity values, Q, using non-parametric tests. We found that, in general, the modularity of observed networks always exceeded that of surrogate networks. Here, for visualization, we show the mean observed (red) and mean surrogate (blue) modularity for all subjects. We note that there were inter-individual differences in modularity. To control for these differences in our visualization we normalized the modularity of both the observed and surrogate data by the mean modularity of the observed network at the first γ value. We note that this normalization – a scaling – was carried out strictly for the sake of visualization and does not influence the results of any statistical comparison.

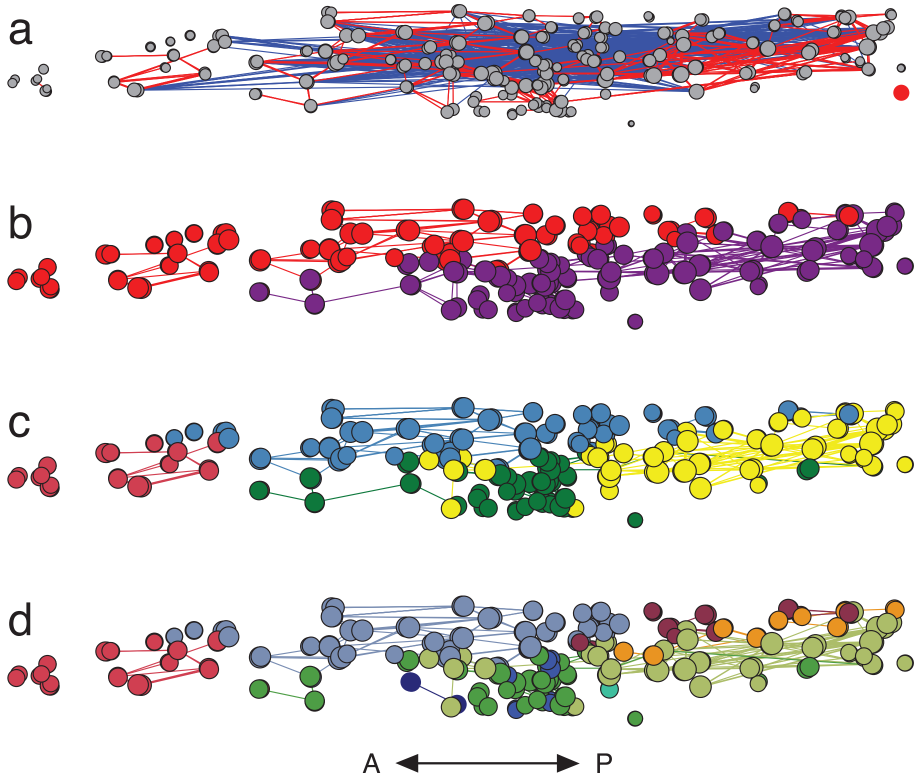

FIG. S4. **Side projection of connectivity and module labels.** (*a*) Side projection of anatomical connectivity shown in Figure. **??**a. (*b*, *c*, *d*) Side projection of module labels with 2, 4, and 9 modules. Originally shown in Figure. **??**b, e, h.

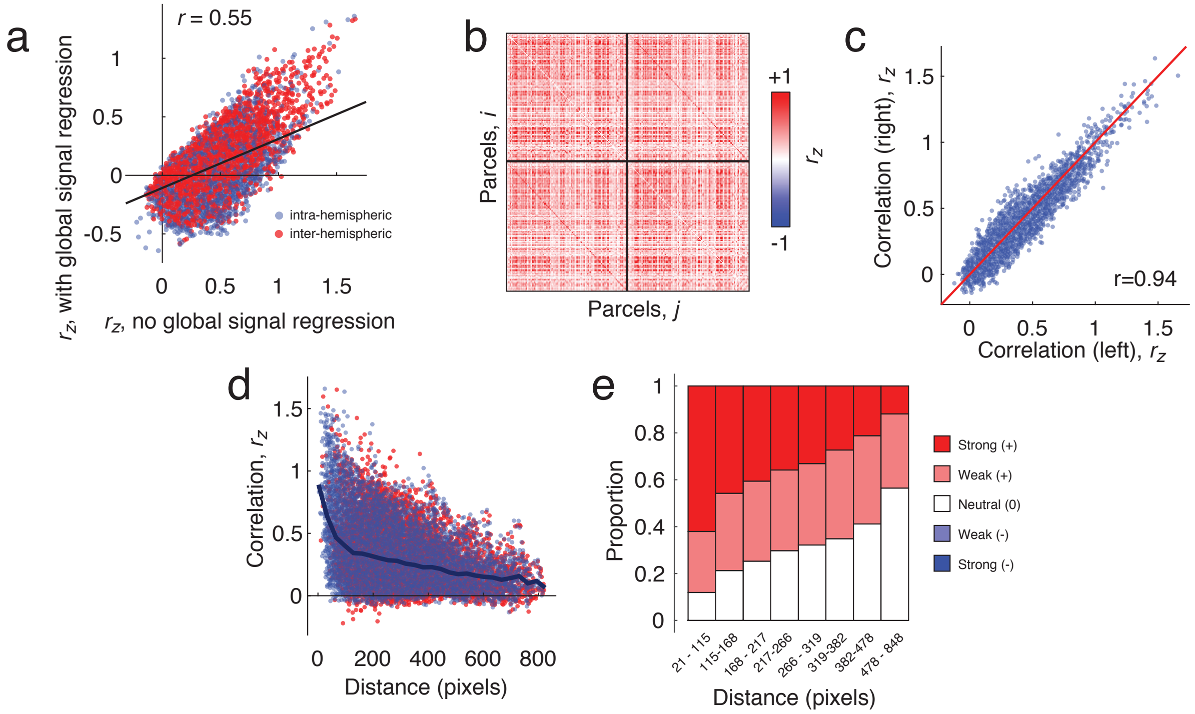

FIG. S5. **Comparison of spontaneous FC with and without global signal regression.** (a) Scatterplot of edge weights from correlation matrices estimated with and without global signal regression. (b) Matrix of parcel-to-parcel correlations based on time series with no global signal regression. (c) Correlation of within-hemisphere connectivity in left and right hemispheres. (d) Scatterplot of correlation magnitude *versus* distance. (e) Correlation magnitudes classified by sign and strength and grouped by distance.

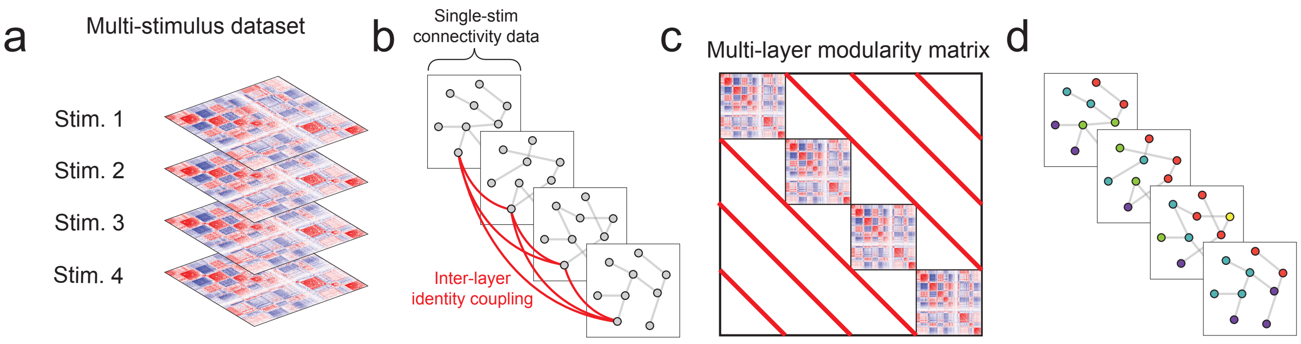

FIG. S6. **Multi-layer modularity maximization.** We used multi-layer modularity maximization to simultaneously detect modules in spontaneous and stimulus-evoked FC. (a) The approach takes as input a series of single-layer connectivity matrices. In this case, the connectivity matrices represent FC estimated under different stimulus or spontaneous conditions. (b) Next, we add weak connections (of magnitude ω) from node i in layer s to itself in all other layers $t \neq s$. (c) Next, we transform the connectivity matrices into single-layer modularity matrices by subtracted from each element the expected connection weight of connections (γ). These single-layer modularity matrices and the inter-layer connections are rearranged into a modularity tensor (shown here in planar form) which is submitted to the modularity maximization algorithm. (d) The multi-layer modularity tensor includes all layers (stimulus conditions) and estimates their modules simultaneously. The result is a set of modules labels that are conserved across layers. That is, a label c that appears in both layers s and t is interpreted as a recurrence of the same module. In summary, multi-layer modularity maximization effectively maps modules from one layer to another; this type of mapping, in general, is not possible using single-layer modularity maximization.

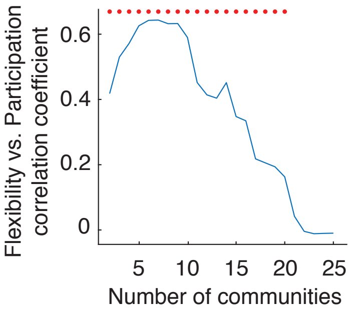

FIG. S7. **Multi-scale correspondence of participation and flexibility.** In the main text, we show that a node's average flexibility (over partitions of the network into different numbers of modules) is correlated with its average participation coefficient. Here, we show that this correspondence is not limited to a particular number of modules, but holds over a broad range. We plot the magnitude of the correlation between flexibility and participation coefficient as a function of the number of modules. Red stars indicate $p < 0.05$.

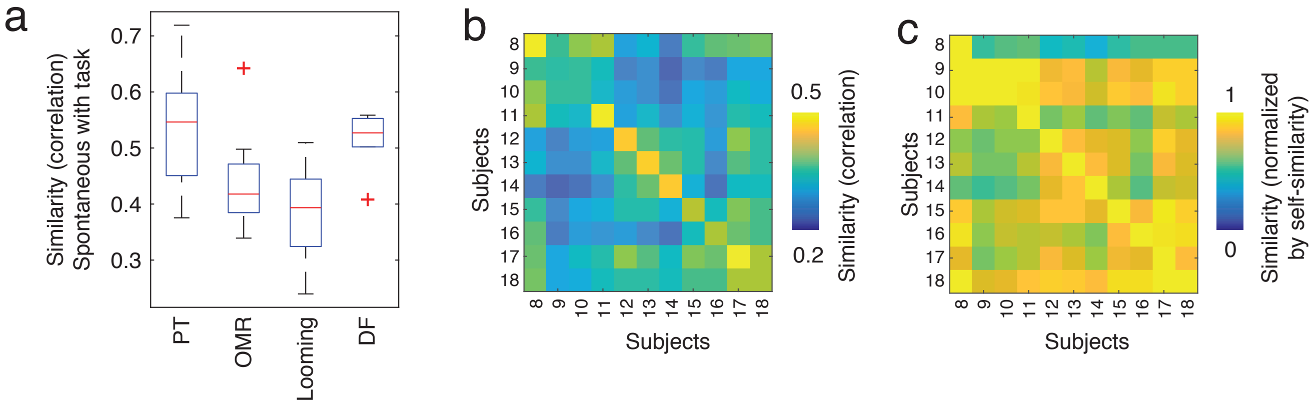

FIG. S8. **Spontaneous to task similarity at the level of individual subjects.** (a) Here, we calculate the similarity (correlation) of connectivity estimated during spontaneous and task conditions. (b) We also calculate the average similarity of all task + spontaneous conditions for each subject to themselves and to every other subject. Note that the diagonal elements are, on average, stronger than the off-diagonal, indicating that subjects to be most similar to themselves, irrespective of task. (c) For the sake of illustration, we divide the elements in every row by the diagonal element. This sets the diagonal element (self-similarity) equal to 1, making it easier to identify, for each row, the strongest elements.

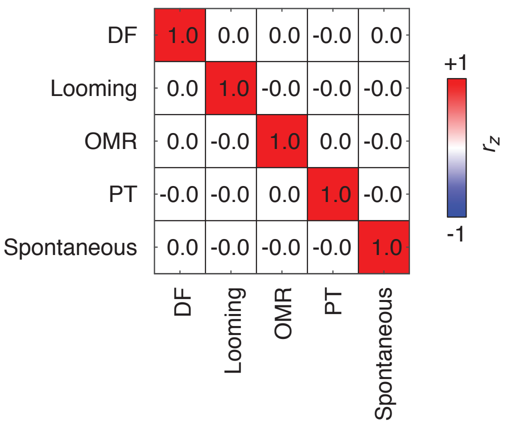

FIG. S9. **Correlation between stimulus-induced and spontaneous FC**. We used the Mantel test [?] to estimate the statistical significance of the correlations between spontaneous FC and stimulus-induced FC. Here, we show the mean correlations of the null distribution. Note that compared to the Fig. 4b in the main text, the off-diagonal correlations are all approximately zero.

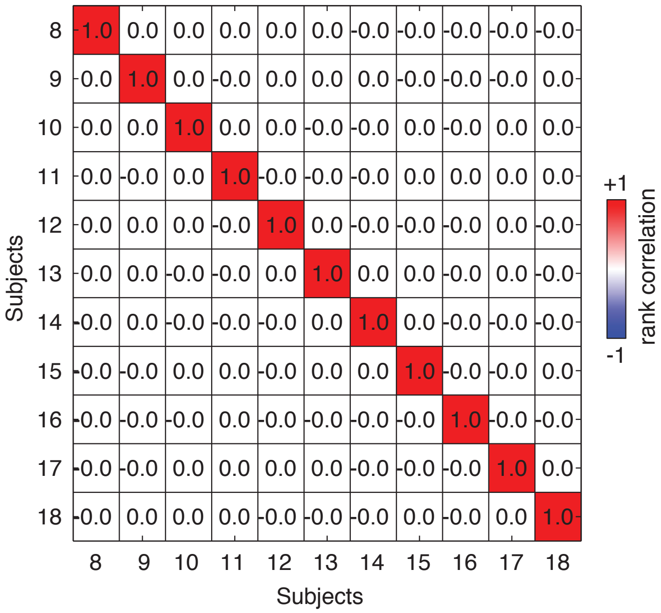

FIG. S10. **Inter-subject similarity from phase-randomized surrogate time series**. We generated a phase-randomized surrogate time series for each subject, estimated FC from these surrogate data, and compared these estimates between all pairs of subjects. We repeated this procedure 1000 times. For all pairs of subjects, the inter-subject similarity was significantly stronger in the real dataset than in the surrogate time series, suggesting that the similarity between subjects cannot be attributed to low-level statistical properties of parcel time series.
